# A Secured Authentication Protocol for Wireless Sensor Networks Using Elliptic Curves Cryptography

**DOI:** 10.3390/s110504767

**Published:** 2011-05-02

**Authors:** Hsiu-Lien Yeh, Tien-Ho Chen, Pin-Chuan Liu, Tai-Hoo Kim, Hsin-Wen Wei

**Affiliations:** 1 Institute of Information System and Applications, National Tsing Hua University, No. 101, Section 2, Kuang-Fu Road, HsinChu, 30013, Taiwan; 2 Department of Computer Science, National Tsing Hua University, No. 101, Section 2, Kuang-Fu Road, HsinChu, 30013, Taiwan; E-Mails: riverchen@rtlab.cs.nthu.edu.tw (T.-H.C.); flash@itri.org.tw (P.-C.L.); 3 Department of Multimedia Engineering, Hannam University, No.133 Ojeong-dong, Daeduk-gu, Daejeon 306-791, Korea; E-Mail: taihoonn@empas.com; 4 Institute of Information Science, Academia Sinica Taipei, Taiwan; E-Mail: hwwei@iis.sinica.edu.tw

**Keywords:** authentication, security, ECC, wireless sensor network

## Abstract

User authentication is a crucial service in wireless sensor networks (WSNs) that is becoming increasingly common in WSNs because wireless sensor nodes are typically deployed in an unattended environment, leaving them open to possible hostile network attack. Because wireless sensor nodes are limited in computing power, data storage and communication capabilities, any user authentication protocol must be designed to operate efficiently in a resource constrained environment. In this paper, we review several proposed WSN user authentication protocols, with a detailed review of the M.L Das protocol and a cryptanalysis of Das’ protocol that shows several security weaknesses. Furthermore, this paper proposes an ECC-based user authentication protocol that resolves these weaknesses. According to our analysis of security of the ECC-based protocol, it is suitable for applications with higher security requirements. Finally, we present a comparison of security, computation, and communication costs and performances for the proposed protocols. The ECC-based protocol is shown to be suitable for higher security WSNs.

## 1. Introduction

As wireless communication technology has matured, the deployment of Wireless Sensor Networks (WSNs) has become more common. Wireless communication is a natural fit for sensor networks for the following reasons: it reduces the cost of infrastructure, allowing sensor networks to be deployed in areas that were once cost prohibitive and it allows a greater range of applications than fixed location sensor networks [1]. WSNs are now providing economical solutions in a host of diverse industries: electric utilities use WSNs for remote voltage monitoring, museums use WSNs for humidity monitoring and control, health care providers use WSNs for patient monitoring and notification, and they are in use in the military. Other applications include environment tracking and habitat monitoring, *etc.* [2–5].

A key requirement for WSN is user authentication [6,7]. The client devices (remote wireless sensor nodes) need to be authenticated before being allowed to join the WSN and have access to the WSN’s resources. To date, most user authentication methods have focused on protocol implementations in the network and link layers. Accordingly, we propose an efficient protocol implementation in the WSN application layer. It should be noted that, in order to limit power consumption by sensor nodes and to overcome limitations in computation capacity, user authentication in a WSN is typically done in dedicated gateway node (GW-node) [8].

Sastry and Wagner [9] proposed a security enhancement using access control lists (ACL’s) in the GW node. In addition to verifying a client’s identity and arranging the nearest sensor node, an ACL would be maintained. The ACL would be limited to 255 entries. Watro *et al.* [10] proposed a complex mathematical method for user authentication employing RSA and Diffie-Hellman algorithms to calculate an encrypted public key (TinyPK authentication), but this protocol is open to hostile attack by a user masquerading as a sensor node (spoofing). Wong *et al.* [11] proposed a less complex, light-weight, dynamic user authentication method using a hash-based protocol. Their method recommended using the security features of the IEEE 802.15.4 MAC sublayer. Das [12] and Tseng *et al.* [13] pointed out that both Watro’s and Wong’s user authentication methods were vulnerable to stolen-verifier, replay, and forgery attacks (made possible by allowing multiple users with a single login ID). Das [12] proposed a two factor method of user authentication. This method is designed to protect against the aforementioned stolen-verifier, replay, and forgery attacks. Tseng *et al.* [13] further pointed out that Wong’s method was vulnerable to stolen passwords and that Wong’s method prevented users from freely changing their password. Tseng *et al.* proposed an enhanced user authentication method that is design to prevent the various attacks and to reduce the vulnerability to stolen passwords. Khan *et al.* [14,15] and Chen *et al.* [16] reviewed the Das two factor method and found additional security issues. Chen *et al.* [16] proposed a more secure and robust two-factor user authentication in WSNs. Unfortunately, we find that the Chen *et al.* proposal fails to provide a secure method for updating user passwords and is vulnerable to the insider attack problem.

To address all of the issues raised in the above studies, we propose a novel user authentication protocol for wireless sensor networks, using Elliptic Curves Cryptography (ECC) and smart cards. Our proposal addresses the key security issues, while at the same time reducing computational load requirements. The remainder of this paper is organized as follows: in Section 2, we review the Das method and perform a detailed cryptanalysis of that method; next we present the ECC-based authentication protocol (EAP) for WSNs in Section 3. In Section 4, we present a security and performance analysis of the related protocols. Then, in Section 5, we provide some concluding remarks.

## 2. Related Works

### 2.1. Review of Das’ Scheme

This section provides a brief review of the Das method and analyzes its protocol. Before this analysis we first summarize in Table 1 the notations used throughout this paper and their corresponding definitions.

Das’ protocol involves the registration phase, login phase and verification phase, and can be briefly described as follows:

(1) Registration phase:

In this phase, a user *U_i_* submits his/her *ID_i_* and *PW_i_* to the GW-node in a secured manner. Then, the GW-node issues a license to *U_i_*. The steps are described as follows:

Step 1: *U_i_* ⇒ GW-node:{*ID_i_*, *PW_i_*}. A *U_i_* enters an identity *ID_i_* and a password *PW_i_* and then sends {*ID_i_*, *PW_i_*} to the GW-node using a secure channel.
Step 2: GW-node ⇒ smart card of *U_i_* :{ *h*(.), *ID_i_*, *N_i_*, *h*(*PW_i_*), *x_a_*}. The GW-node computes *N_i_* = *h*(*ID_i_* || *PW_i_*) ⊕ *h*(*K*) after receiving the registration request. Then, the GW-node personalizes the smart card with parameters {*h*(.), *ID_i_*, *N_i_*, *h*(*PW_i_*), *x_a_*}. *U_i_* receives the smart card information using a secure channel.

(2) Login phase:

When user *U_i_* enters an *ID_i_* and a *PW_i_* in order to carry out some inquiry or to access data from the WSN, the smart card must confirm the validity of *U_i_* according to the following steps:

Step 1: Validate *U_i_*. The entered *ID_i_* and *PW_i_* are validated against the *ID* and *PW* stored on the user’s smart card. If *U_i_*’s identification validation fails, the smart card will terminate this request.
Step 2: *U_i_*’s smart card calculates *DID_i_* and *C_i_*.
  *DID_i_* = *h*(*ID_i_* || *PW_i_*)⊕*h*(*x_a_*|| *T*), where *T* is the login system timestamp.
  *C_i_* = *h*(*N_i_* || *x_a_* || *T*).
Step 3: *U_i_*→GW-node:{*DID_i_*, *C_i_*, *T*}.
  {*DID_i_*, *C_i_*, *T*} is transmitted to the GW-node via public channel.

(3a) Verification phase (gateway node):

When the GW-node receives a login request {*DID_i_*, *C_i_*, *T*} at time T*, the GW-node performs the following steps to verify the identity of *U_i_*:

Step 1: Validates if T*–T < Δ*T*.
  If (T* – *T*) ≤ Δ*T* then the validity of *T* can be certain, and the GW-node proceeds to the next step. Otherwise, the GW-node rejects the request. Here, Δ*T* denotes the expected time interval for transmission delay.
Step 2: Calculates $C_{i}^{*}$.
  *h*(*IDi* || *PW_i_*)* = *DID_i_*⊕*h*(*x_a_* || *T*)
$C_{i}^{*}\;=\;h(h(ID_{i}\;||PW_{i})*\;||h(K)\;||x_{a}||T)$.
Step 3: Confirms whether the $C_{i}\;=\;C_{i}^{*}$.
  If the $C_{i}\;=\;C_{i}^{*}$, then the GW-node accepts the login request and sends a request to *Sn*.
Step 4: GW-node→*S_n_*:{*DID_i_*, *A_i_*, *T'*}.
The GW-node calculates *A_i_* = *h*(*DID_i_* || *S_n_* || *x_a_* || *T'*) and transmits a request {*DID_i_*, *A_i_*, *T'*} to *S_n_* over a public channel. *T'* is the GW-node request timestamp. *A_i_* is generated using the *x_a_* parameter, thus the value of *A_i_* can be used by *S_n_* to ensure that the message originates from a valid GW-node.

(3b) Verification phase (sensor node):

When *S_n_* receives request {*DID_i_*, *A_i_*, *T'*} at time *T*, *S_n_* performs the following steps to verify the validity of the request:

Step 1: Validates if *T* – *T’* < Δ*T*.
  If (*T* – *T’*) ≤ Δ*T* then the validity of *T'* can be certain, and *S_n_* proceeds to the next step.
Step 2: Recalculates *A_i_*.
  *A_i_* = *h*(*DID_i_* || *S_n_* || *x_a_* || *T'*)
Step 3: Confirms whether the value of the locally calculated *A_i_* is the same as the value of *A_i_* in the GW-node request.
If the value of the locally calculated *A_i_* is the same as the value of *A_i_* in the GW-node request, then *S_n_* responds to *U_i_*’s original request. Otherwise, *S_n_* rejects the request.

### 2.2. Cryptanalysis of Das’ Protocol

Recently, several studies have analyzed security flaws in Das’ scheme [14–16]. In this section, we also discuss the requirements of security in WSNs and describe the primary flaw of Das’ protocol (it omits mutual authentication) and several secondary security issues [14–16].

#### 2.2.1. Security Requirements in Wireless Sensor Networks

Sastry and Wagner [9] noted several problems with regard to the security of user authentication provided by IEEE 802.15.4 [17]. They cited ACL management problems, loss of ACL state due to power interruptions, and key management problems. They concluded that IEEE 802.15.4 provides insufficient user authentication security and provided some solutions for the noted problems. However, above and beyond the security issues noted by Sastry and Wagner, there are two additional security issues that must be addressed:

Secure user authentication in WSNs should include, to the extent possible, methods for addressing application layer issues such as masquerade, replay, and forgery attacks.
Secure user authentication in WSNs should be based on mutual authentication.

#### 2.2.2. No Mutual Authentication

Because Das’ protocol does not provide mutual authentication [14–16], a malicious user can attack a WSN that uses the Das protocol by means of eavesdropping and masquerading. The attack could be accomplished as follows:

- *U_i_* sends the message {*DID_i_*, *C_i_*, *T*} to the GW-node for accessing the WSN.
- The GW-node sends the message {*DID_i_*, *A_i_*, *T*} to *S_n_* for asking the service for *U_i_*.
- The attacker captures the message {*DID_i_*, *A_i_*, *T*} via eavesdropping.
- The attacker provides an *S_M_* which masquerades as *S_n_* to get the *U_i_*’s request data or hold back the request.
- Since *S_M_* co-works with *U_i_* continuously, the *U_i_* access requests will continue to fail.

With the Das method, after accepting the login request of *U_i_*, the GW-node sends a message {*DID_i_*, *A_i_*, *T’*} to some nearest sensor node *S_n_*. Here the value of *A_i_* is computed by *A_i_* = *h*(*DID_i_* || *S_n_* || *x_a_* || *T’*), where *T'* is the current timestamp of GW-node. The value of *A_i_* is used to assure the sensor node that the message has come from the real GW-node. The GW-node message directs the sensor node to reply to the query with the data which *U_i_* has requested. However, there is no mechanism for the GW-node to be assured that the reply message was initiated from the queried sensor node. Thus, the Das-scheme only provides unilateral authentication between the GW-node and sensor node. There is no mutual authentication between the two nodes.

#### 2.2.3. No Protection against Insider Attacks

Nowadays users use a single common password for accessing different applications or servers. The situation is common practice and this is done for their convenience. It relieves the user from having to remember multiple passwords. Nevertheless, if the system manager or a privileged user of the GW-node obtains the common password of *U_i_*, he/she may try to impersonate *U_i_* by accessing other servers where *U_i_* could be a registered user. In the Das scheme [14,15], *U_i_* performs registration with the GW-node by presenting a password in plain format. Thus, the Das protocol does not provide sufficient protection against an insider attack on a GW-node by a privileged user.

#### 2.2.4. No Provision for Changing/Updating Passwords

The fixed password is definitely suffered from threats than an updating password. It is a widely recommended security policy, for highly secure applications, that users should update or change their passwords frequently. In the scheme [14,15], there is no provision for a user to easily change his/her password.

#### 2.2.5. No Protection against Forgery Attacks

A legal user of the system can launch a forgery attack against the WSN by eavesdropping and masquerading. A forgery attack can be launched as follows [16]:

- A legal user of the system U* can login to the WSN at *T_A_* and *T_B_* accurately.
- Suppose U* has embedded a synchronized Trojan virus into legal user *U_i_*’s system.
- When *U_i_* wants to login to the WSN at *T_A_* and *T_B_*, U* can eavesdrop on the messages {*DID_i_*, *C_i_*, *T_A_* } and {*DID_i_*, *C_i_*, *T_B_* } between the GW-node and *U_i_* at *T_A_* and *T_B_*. To judge which message is *DID_i_* or *C_i_* as follows:
  Step 1.U* can obtain the following messages: *DID_i_*(*T_A_*) = *h*(*ID_i_* || *PW_i_*)⊕*h*(*x_a_* || *T_A_*) and *DID_i_*(*T_B_*) = *h*(*ID_i_* || *PW_i_*)⊕*h*(*x_a_* || *T_B_*).
  Step 2. And then U* can forge the dynamic login identity DID*(*T_A_*) and DID*(*T_B_*).
    DID*(*T_A_*) = *h*(ID* || PW*)⊕*h*(*x_a_* || *T_A_*)
    DID*(*T_B_*) = *h*(ID* || PW*)⊕*h*(*x_a_* || *T_B_*).
- U* can use the login phase formula to compute *DID_i_*(*T_B_*), where *DID_i_*(*T_B_*) is calculated as *DID_i_*(*T_B_*) = *DID_i_*(*T_A_*)⊕DID^*^(*T_A_*)⊕DID^*^(*T_B_*) = *h*(*ID*_*i*_‖*PW_i_*) ⊕ 
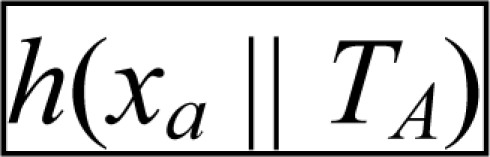
 ⊕ 
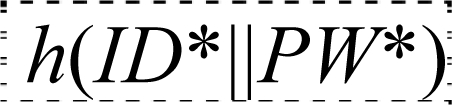
⊕ 
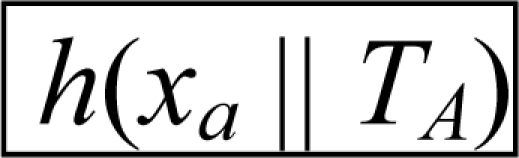
 ⊕ 
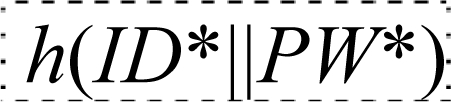
 ⊕ *h*(*x_a_*‖*T*_*B*_)
- After U* obtains *U_i_*’s *DID_i_*(*T_B_*), U* sends a new session message {*DID_i_*(*T_B_*), *C_i_*, *T_S_*} within ΔT timestamp for a new login request. The timestamp *T_S_*, where *T_S_* = *T_B_*, is made by U* for attack on the WSN.
- Thus, the GW-node will verify message {*DID_i_*(*T_B_*), *C_i_*, *T_S_* } from U* with following steps: U*→GW-node:{*DID_i_*(*T_B_*), *C_i_*, *T_S_*}
  Step 1. The GW-node receives {*DID_i_*(*T_B_*), *C_i_*, *T_S_*} at T* and checks for T* – *T_S_* < Δ*T*. The GW-node passes the verification and proceeds to the next step.
  (T* – *T_B_* < Δ*T* is known and *T_S_* = *T_B_* was made arbitrarily by U*)
  Step 2. The GW-node calculates *h*(*ID_i_* || *PW_i_*)* = *DIDi*(*T_B_*) ⊕ *h*(*x_a_* || *T*) and obtains $C_{i}^{*}\;=\;h(h(ID_{i}\;||PW_{i})*||h(K)||x_{a}||T)\;(C_{i}^{*}=C_{i})$ to pass the verification and proceed to the remaining steps.

Consequently, the Das protocol does not provide sufficient protection against a forgery attack by a legal user.

## 3. ECC-Based Authentication Protocol (EAP) for WSN

**Figure 1.**
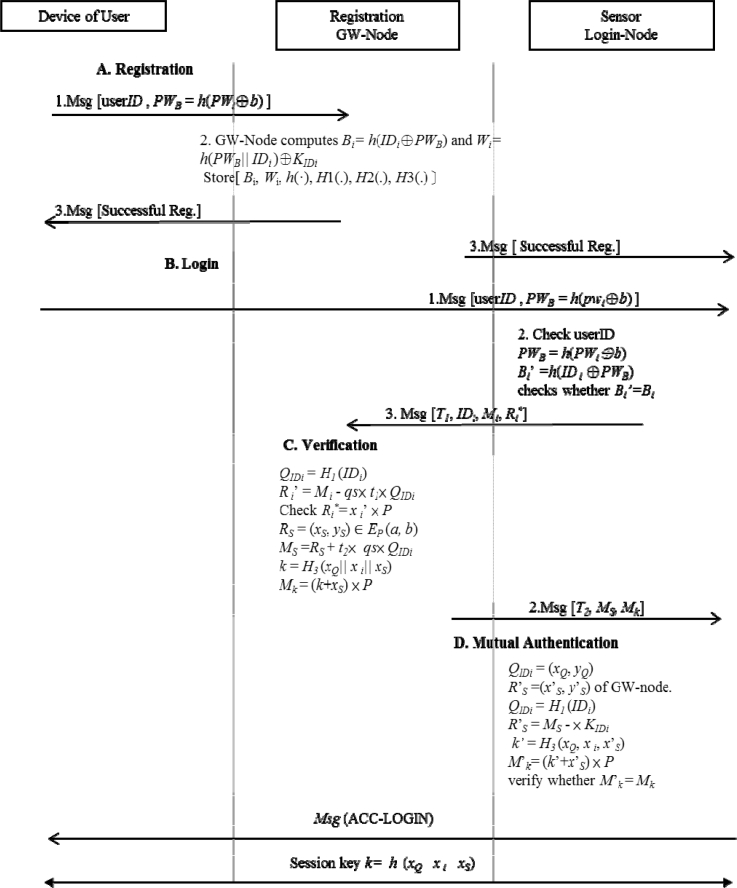
Communication handshakes of the proposed scheme.

This section proposes a more efficient authentication mechanism using ECC. First, we review the fundamentals of Elliptic Curves and then survey the Elliptic Curves Cryptography (ECC) which is suitable for our construction of a secured authentication protocol for wireless sensor networks. The proposed five phases will be described later. The overall handshake of the proposed protocol is illustrated in Figure 1. The GW-node, *S_n_* and user use the *h*(*x_Q_*||*x_i_*||*x_S_*) as a session key with communication handshakes.

### 3.1. ECC Based Authentication Protocol

In 1985 Miller and Kobiltz proposed a secure and efficient elliptic curve cryptosystem (ECC) [17,18]. Because ECC provides a smaller key size than any other cryptosystem, it is suitable for application in smart card and wireless systems.

An elliptic curve is a cubic equation of the form: E: *y*^2^ + *axy* + *by* = *x*^3^ + *cx*^2^ + *dx* + *e*, where *a*,*b*,*c*, *d*,*e* are real numbers. With regard to cryptography, we focus on the finite field of ECC and aim mainly at the prime *p* of elliptic curve group. The mathematical equation of ECC satisfies the form: *E : y*^2^ = (*x*_3_ + *ax* + *b*) mod *p*, where (4*a*^3^ + 27*b*^2^) ≠ 0. Let *F_p_* denote the finite field of points, where *p* is a large prime number and containing *x*, *y*, *a*,*b* elements. The equation points and the point at infinity ***O*** compose the elliptic curve group over real numbers. We find a large prime number *n* such that *n* × *P* = ***O*** using the elliptic curve addition algorithm. Here, × denotes an elliptic curve multiplication. The arithmetic of elliptic curve discrete logarithm problem (ECDLP) is given points *Q* and *P*, where *Q*, *P*∈ *F_p_* and are both publicly known, determine the random number *K*, *0* < *K* < *n-1*, and compute *Q* as : *Q* =*K*×*P* is satisfies. It is hard to determine *K* given *Q* and *P*, namely, ECDLP is a complex mathematical problem such that the security is achieved. The analog of Diffie-Hellman key exchange uses elliptic curve characteristics to complete key exchange. The key exchange between *U_A_* and *U_B_* can be done as follows [18–20]:

- The user *U_A_* chooses a random integer *r_A_* as a private key*,* where *r_A_* *< n* and computes the public key *Q_A_* as: *Q_A_* = *r_A_* × *P*. Then, *U_A_* sends *Q_A_* to the user *U_B_*.
- The user *U_B_* selects a random integer *r_B_* as a private key*,* where *r_B_* *< n* and computes the public key *Q_B_* as: *Q_B_* = *r_B_* × *P.U_B_* sends *Q_B_* to *U_A_*.
- *U_A_* can compute shared key *K_A_* *= r_A_* × *Q_B_* = *r_A_* × *r_B_* × *P* and *U_B_* can compute shared key *K_B_* *= r_B_* × *Q_A_* = *r_B_* × *r_A_* × *P*. In this manner we find *K_A_* *= K_B_*.

### 3.2. Registration Phase

This phase is invoked whenever user *U_i_* performs registration with the WSN. Then, *U_i_* submits {*ID_i_*, *PW_B_*} to the GW-node by the secured channel. Then, the GW-node performs the license to *U_i_*. The following steps are performed to complete this phase:

Step 1: *U_i_* ⇒ GW-node:{*ID_i_*, *PW_B_*}.
  *U_i_* chooses his/her *ID_i_* and *PW_i_* password and randomly chooses a large number *b* for computing *PW_B_* *= h*(*PW_i_* ⊕ *b*).
  Step 2: After receiving the registration request, the GW-node computes *K_IDi_* = *qs* × *H_1_*(*ID_i_*) ∈ *G_p_*_,_ where *K_IDi_* is *U_i_*’s authentication key and *G_p_* denotes a cyclic addition group of *P*.
  Step 3: GW-node selects a base point *P* with the order *n* over *E_p_*(*a*, *b*), where *n* is a large number for the security considerations. Then, the GW node derives its private/public key pair (*q_s_*, *Q_S_*) by computing *Q_S_* = *q_s_* × *P*. (Here × denotes an elliptic curve multiplication).
  Step 4: GW node computes *B_i_* *= h*(*ID_i_*⊕*PW_B_*) and *W_i_* *= h*(*PW_B_* *|| ID_i_*)⊕*K_IDi_*.
  Step 5: GW-node ⇒ smart card of *U_i_* :{*B_i_*, *W_i_*, *h*(·), *b*, *H_1_*(.), *H_2_*(.), *H_3_*(.)}.
  GW-node stores {*B_i_*, *W_i_*, *h*(·), *H_1_*(.), *H_2_*(.), *H_3_*(.)} on a smart card and sends the smart card to *U_i_* over a secure channel. Here *H_1_*(.), *H_2_*(.) and *H_3_*(.) are one-way hash functions, *H_1_*(.): {0, 1} →*G_p_*, $H_{2}(.):\{0,1\}\;\to Z_{P}^{*}$ and $H_{3}(.):\{0,1\}\;\to Z_{P}^{*}$.
Step 6: Upon *U_i_* receiving the smart card, *U_i_* stores the random number *b* in the smart card. Such that the smart card contains {*B_i_*, *W_i_*, *h*(·), *b*, *H_1_*(.), *H_2_*(.), *H_3_*(.)}.

### 3.3. Login Phase

Assume that *U_i_* enters in order to ask a service from the network, the smart card must perform the following steps to validate the legality of *U_i_*:

Step 1: *U_i_* enters his/her *ID_i_* *and PW_i_* to login to obtain the message for GW-node request.
Step 2: *U_i_* computes *PW_B_* *= h*(*PW_i_⊕b*) and *B_i_*’ = *h*(*ID_i_*⊕*PW_B_*) and checks whether *B_i_*’ = *B_i_*. If it holds, *U* *_i_* computes *Q* = *h*(*PW_B_||ID_i_*) and *K_IDi_* = *W_i_*⊕*Q.*

When the login request has been accepted, the user proceeds with the remaining steps:

Step 1: After *U_i_* obtaining his/her authentication key *K_IDi_*, *U_i_* chooses a random point *R_i_* = (*x* *_i_*, *y_i_*) ∈ *E_P_* (*a*, *b*), where *x* *_i_* and *y* *_i_* are *x* and *y* coordinating point of *R_i_*.
Step 2: *U_i_* computes *t*_1_ = *H*_2_ (*T*_1_), *M_i_* =*R_i_* + *t_1_* × *K_IDi_* and $R_{i}^{*}\;=\;x_{i}\;\times P$ at the timestamp *T_1_*.
Step 3: *U_i_*→GW-node: {*T_1_*, *ID_i_*, *M_i_*, $R_{i}^{*}$}.
  *U_i_* sends message *Msg*(*T_1_*, *ID_i_*, *M* *_i_*, $R_{i}^{*}$) to GW-node.

### 3.4. Verification Phase

After receiving the login request message *Msg*(*T_1_*, *ID_i_*, *M_i_*, $R_{i}^{*}$) at *T_1_* through the nearest sensor node (*S_n_*), the GW-node executes the following steps to verify the user *U_i_*:

Step 1: Compute *Q_IDi_* and *R_i_*’
  GW-node performs the following computations to obtain *Q_IDi_* *=* (*x_Q_*, *y_Q_*) and *R_i_*’ = (*x_i_*’, *y_i_*’) of *U_i_*.
  *QIDi* = *H1* (*IDi)*
  *t*_1_ = *H*_2_ (*T*_1_)
  *R_i_*’ = *M*_*i*_ – *qs*× *t*_*1*_× *Q_IDi_*
Step 2: The GW node verifies whether $R_{i}^{*}\;=\;x_{i}^{'}\;\times P$. If it holds, *U_i_* is authenticated by GW-node.
Step 3: GW-node→ *U_i_*: {*T_2_, M_S_, M_k_*} through *S_n_*.
  The GW node chooses a random point *R_S_* = (*x_S_*, *y_S_*) ∈ *E_P_* (*a, b*) and computes *t*_2_ = *H*_2_ (*T*_2_), *M_S_* =*R_S_* + *t_2_*× *qs*× *Q_IDi_*, session key *k = H_3_* (*x_Q_* *|| x_i_* *|| x_S_*) and *M_k_* = (*k* + *x_S_*) × *P*.

GW-node sends a message *Msg*(*T_2_, M_S_, M_k_*) through the public channel in order to respond to the request of *S_n_* at the timestamp *T_2_*.

### 3.5. Mutual Authentication Phase

The GW-node sends *Msg*(*T_2_, M_S_, M_k_*) to the *S_n_* and then *S_n_* sends *Msg*(ACC-LOGIN) to the GW-node. The steps are described as follows:

Step 1: Compute *Q_IDi_* and *R*’*_S_*
  After receiving *Msg(T2, MS, Mk),* the *S_n_* execution obtains the following computation
  *QIDi* = (*x_Q_, y_Q_*) and *R’_S_* = (*x’_S_, y’_S_*) of the GW-node.
  *Q_IDi_* = *H_1_* (*ID_i_*)
  *t*_2_ = *H*_2_ (*T*_2_)
  *R*’*_S_* = *M_S_* – *t*_2_ × *K_IDi_*
Step 2: *S_n_* computes *k’* = *H_3_* (*x_Q_|| x_i_* *|| x*’*_S_*) and *M*’*_k_*= (*k*’ + *x*’*_S_*) × *P* to verify whether *M*’*_k_* = *M_k_*. If it holds, GW-node is successfully authenticated by *S_n_*.

### 3.6. The Password-Changing Phase

When a user *U_i_* enters an *ID_i_* and a *PW_i_* in order to request a password change, the smart card must compute a new value of $PW_{B}^{*}\;=\;h(PW_{i}^{*}⊕b)$ to the GW-node. After receiving the password change request, the GW-node computes $B_{i}^{*}$ and $W_{i}^{*}$.

Step 1: GW-node computes $B_{i}^{*}\;=\;h(ID_{i}⊕PW_{B}^{*})$ and $W_{i}^{*}\;=\;h(PW_{B}^{*}||ID_{i})⊕K_{IDi}$.
Step 2: GW-node stores the new vale on smart card.
The smart card replaces the original values of *B_i_*, *W_i_* with the new value $B_{i}^{*}$, $W_{i}^{*}$ and $PW_{B}^{*}\;=\;h(PW_{i}^{*}⊕b)$.

## 4. Security and Performance Analysis

### 4.1. Security Analysis

The studies we have referenced in this paper have discussed the security issues of remote user authentication. Below is a summary of those security issues, along with the reasons we believe our proposed ECC protocol can address those issues.

**Resistance to insider attack:** It is common practice for users to apply the same common password to access different applications. If a privileged insider has knowledge of another user *U_i_*’s password, it hey may try to impersonate user *U_i_* to access network applications. Our proposed protocol registers user *U_i_* using cipher code *PW_B_* = *h*(*PWi*⊕*b*) over a secure channel. This provides protection against stolen passwords. Thus, our protocol resists insider attacks.

**Resistance to masquerade attack:** To successfully complete a masquerade attack, an attacker must know *U_i_*’s password in order to pass verification in the login phase and to be able to interpret the verification message correctly for mutual authentication. An attacker, even a legitimate user U*, cannot masquerade as a different legitimate user *U_i_* without *U_i_*’s password for forging the messages sent to the GW-node.

**Mutual authentication:** Mutual authentication is an important feature for a verification service that is resistant to server spoofing attacks. Our protocol provides a mutual authentication between the user *U_i_* and the GW-node by using ECC-based public and private keys exchange.

**Securely change/update password:** There is provision for users to update or change their password in our proposed scheme. Namely, a user can send a new password to the GW-node and then the GW-node computes new value of $B_{i}^{*}$, $W_{i}^{*}$ and stores them on the smart card.

We recall that the protocol [12–16] of Wong *et al*. does not provide for mutual authentication, and can be vulnerable to forgery and replay attacks. Besides, the proposal of Watro *et al.* has security weaknesses against masquerade attacks, and Das’ protocol does not provide mutual authentication with an authenticated procedure using the hash function. Further, the weaknesses of Das’ scheme are that it may suffer from an insider attack and a forgery attack. Chen *et al*.’s scheme is similar in Das’ scheme, and also has the insider attack problem. Besides, the referenced proposals all fail to provide a secure method for updating user passwords. Table 2 compares our proposed protocol with the other referenced protocols in terms of protection against attacks. When compared against each other, our protocol provides a solution for user authentication that is more secure than the other referenced protocols.

### 4.2. Performance Analysis

For comparing performance between our protocol and related protocols, we estimate the computation costs. In the definition of computation costs, we define the notation t_h_ as the hash computation time, t_PA_ as the elliptic curve point addition computation time, t_PM_ as the elliptic curve point multiplication computation time, t_E_ as the elliptic curve polynomial computation time, t_PR_ as the private key computation time, and t_PU_ as the public key computation time. Note that the computation costs of t_PU_ and t_PR_ are considerably higher than t_h_ (t_PU_ ≫ t_h_ and t_PR_ ≫ t_h_) because t_PU_ and t_PR_ usually need polynomial computation cost to obtain the public and private keys. Obviously, t_E_, t_PA_, t_PM_ calculates a cubic equation at most and t_h_ calculates a linear equation or quadratic equation at most. The comparison of related protocols is illustrated in Table 3.

When considering the computation cost in the authentication phase (which includes the verification and mutual authentication phases), our protocol requires only 11 t_h_ + 4 t_PA_ + 6 t_PM_ + 2 t_E_. That is, our protocol needs one point addition operation, four point multiplication operations and one polynomial operation in ECC. However, Watro *et al.*’s protocol needs two hash functions and four polynomial computations for private key and public key computation. It uses complex RSA and Diffie-Hellman algorithms for user authentication. The polynomial computation time calculates a prime exponential function which is considerably higher than cubic equation [12,17]. In addition, Watro *et al*.’s protocol needs four polynomial computations, for t_PR_ and t_PU_, are more than the other referenced protocols [12–16]. Besides, our proposed protocol is computed through combination of point addition and point multiplication, point multiplication is defined by repeated addition. Considering the computation costs, ECC can generate smaller key sizes but maintain equivalent levels of security with RSA [18–20]. This is the reason the ECC-based protocol is more practical than Watro *et al*.’s protocol.

Lastly, when considering the communication cost, the proposed protocol has higher computation cost than other protocols, except for Watro *et al*.’s protocol. However, the protocol of Das does not provide mutual authentication. The method we propose solves most of the Das method problems. Furthermore, although Das’s scheme needs five hash computation operations, Wong’s needs four hash computation operations and Chen *et al*.’s protocol performs wireless sensor networking using seven t_h_, their protocols suffer from security issues. Our proposed protocol addresses these issues and provides better security than the other related protocols.

## 5. Conclusions

In this paper, we have analyzed Das’ scheme for user authentication in WSNs. The Das protocol, which does not provide mutual authentication, is susceptible to insider and forgery attacks. We have also reviewed the protocols of Wong *et al*., which is vulnerable to forgery and replay attacks, of Watro *et al*., which is vulnerable to masquerade attacks, and Chen *et al*.’s protocol, which is susceptible to insider attacks. Additionally, a user cannot change his/her password with the former schemes. Since WSNs needs more efficient methods to perform mutual authentication in an insecure network environment, we use an ECC-based mechanism to accomplish this. The proposed protocol can prevent all the problems of the former schemes and provide mutual authentication to protect inside security and outside security. Furthermore, it not only inherits the merits of ECC-based mechanism but also enhances the WSN authentication with higher security than other protocols. Therefore, the proposed protocol is more suited to WSNs environments.

## Figures and Tables

**Table 1.** Notations.

**Symbol**	**Definition**
U	A user
ID	A user’s identity
PW	A user’s password
DID	A user’s dynamic login identity
GW-node	Gateway node of WSN
S_n_	Nearest sensor node of WSN
h(.)	A secure one-way hash function
x_a_	A permanent secret parameter generated securely by the GW-node and stored in some defined sensor nodes before deploying the WSN
K	A symmetric key of GW-node which shared between the GW-node, users and the sensor nodes
||	A string concatenation operation
⊕	A string XOR operation
⇒	A secure channel
→	A public channel

**Table 2.** Security comparison among the referenced protocols.

**Item**	**Proposed**	**Chen *et al.*’s**	**Das’**	**Watro *et al.*’s**	**Wong *et al.*’s**

Avoiding insider attack	Yes	No	No	Yes	Yes
Securely change/update password	Yes	No	No	No	No
Avoiding forgery attack	Yes	Yes	No	Yes	No
Mutual authentication	Yes	Yes	No	Yes	No
Avoiding masquerade attack	Yes	Yes	Yes	No	Yes
Avoiding replay attack	Yes	Yes	Yes	Yes	No
Avoiding guessing attack	Yes	Yes	Yes	Yes	Yes

**Table 3.** Performance comparison among related protocols.

	**Proposed**	**Chen *et al.***	**Das**	**Watro *et al.***	**Wong *et al.***

Authentication (Verification and Mutual Authentication)	11 t_h_ + 4 t_PA_ + 6 t_PM_ + 2 t_E_	7 t_h_	5t_h_	2t_h_+2t_PR_+2t_PU_	4t_h_
